# Supplementary material for: Epithelial, metabolic and innate immunity transcriptomic signatures differentiating the rumen from other sheep and mammalian gastrointestinal tract tissues
Source: PeerJ. 2016 Mar 8;4:e1762. doi: 10.7717/peerj.1762 (PMC4793311; doi:10.7717/peerj.1762)
Supplement: Supplemental Information 2 [file peerj-04-1762-s002.pdf]

Supplementary Figure S1

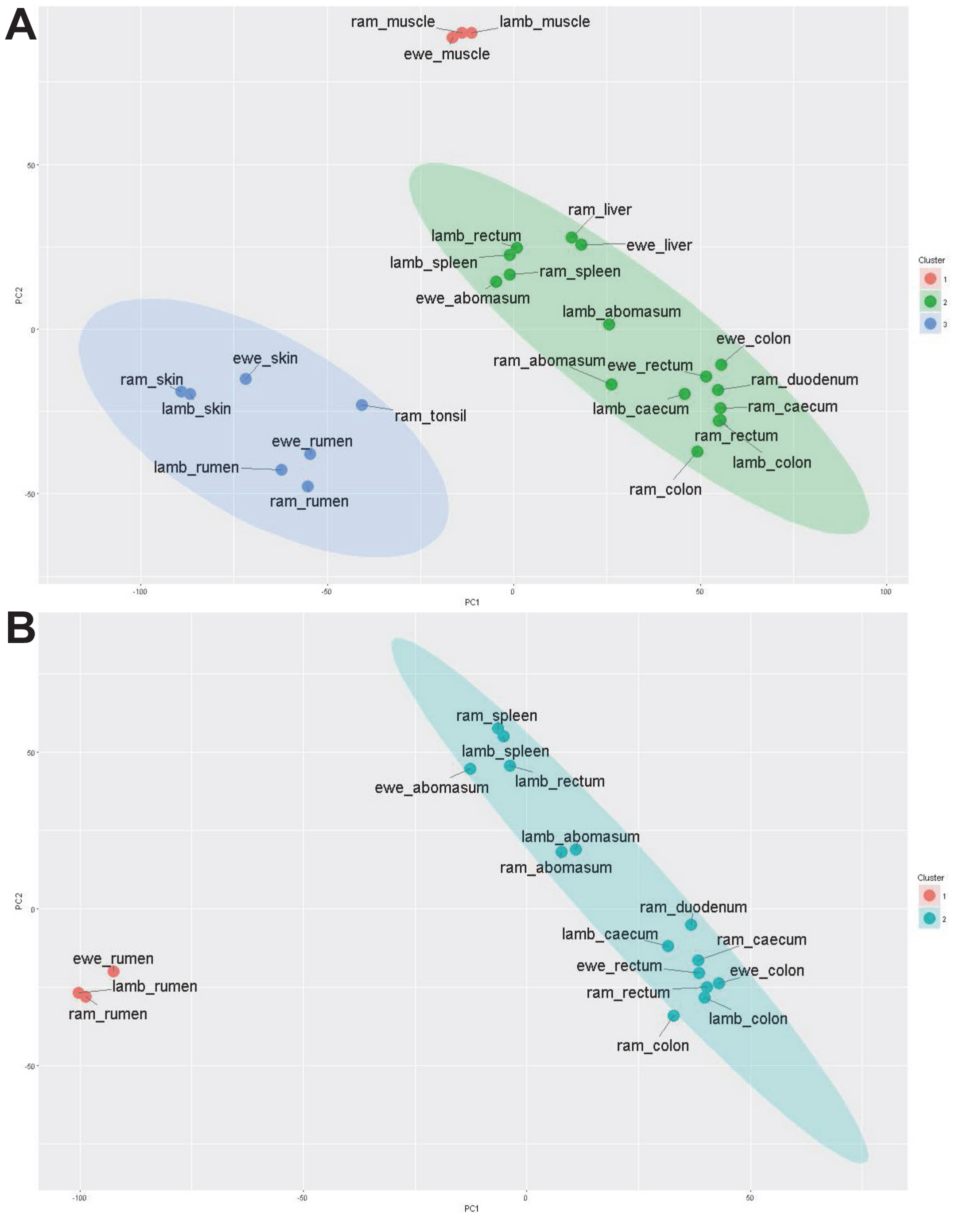

Figure S1. Raw output of PCA clustering of tissues. **A**: Three significantly separated clusters of total 26 studied tissues. **B**: Two significantly separated clusters of studied tissues without epithelial (skin and tonsil) and reference (muscle and liver) tissues. Cluster boundary significance testing is performed based on K-mean clustering and bivariate t-distribution using stat_ellipse package (https://github.com/JoFrhwld/FAAV/blob/master/r/stat-ellipse.R) and presented in ggplot2 in R.

Supplementary Figure S2

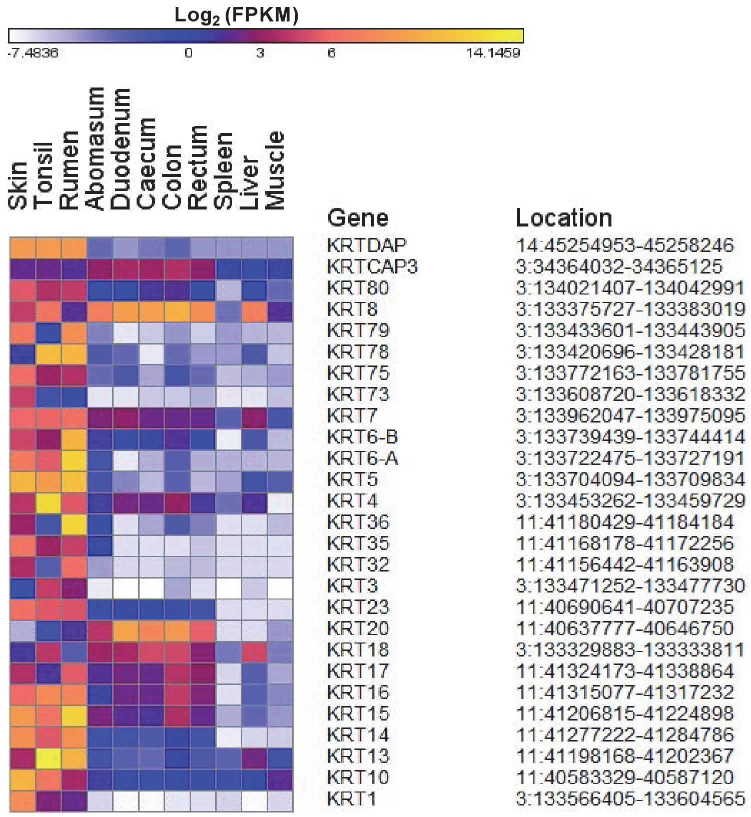

Figure S2. Expression profile of gastrointestine related keratin genes. Keratin genes are selected based on their average count per million values in gastrointestinal tissues > 3.

Supplementary Figure S3

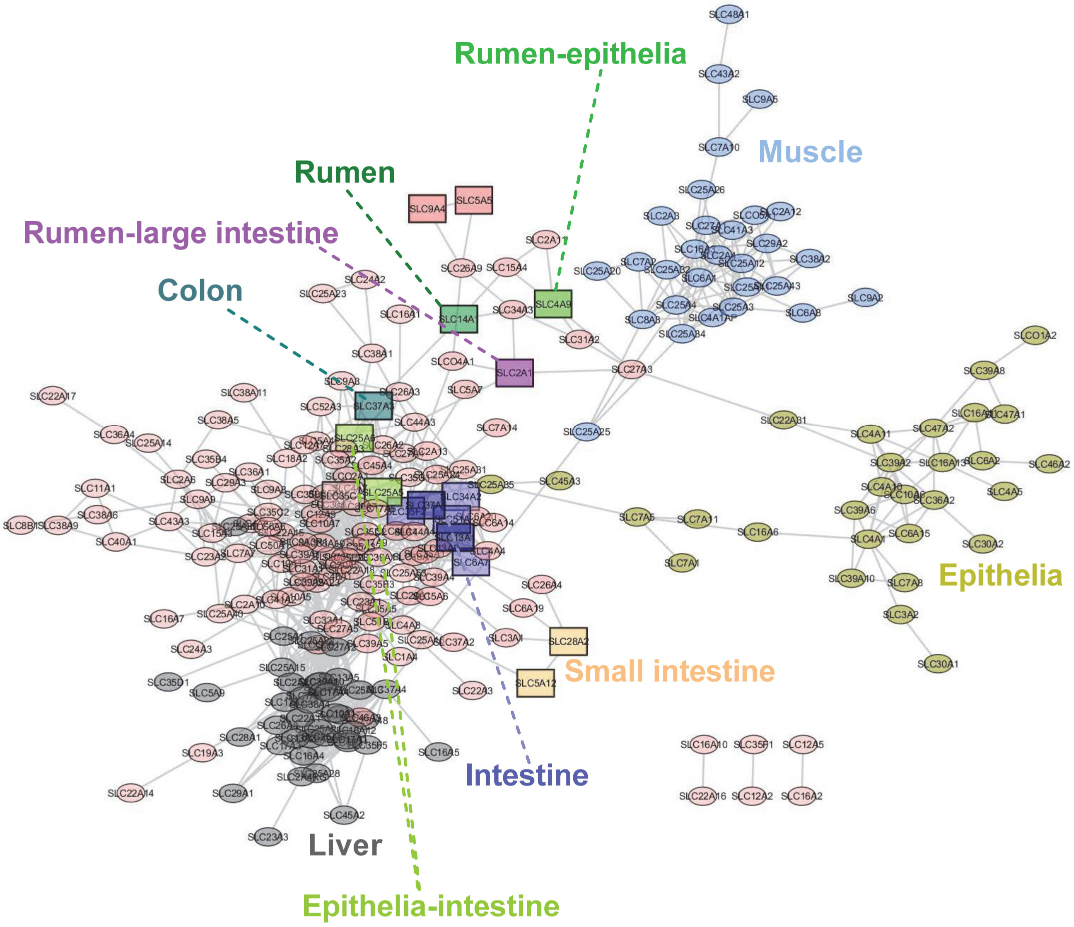

Figure S3. Clustering of transporter genes. Genes in boxes are assigned with sheep clusters as shown in table S1.
